# Medium term effects of a ketogenic diet and a Mediterranean diet on resting energy expenditure and respiratory ratio

**DOI:** 10.1186/1753-6561-6-S3-P37

**Published:** 2012-06-01

**Authors:** Antonio Paoli, Keith Grimaldi, Antonino Bianco, Alessandra Lodi, Lorenzo Cenci, Andrea Parmagnani

**Affiliations:** 1 Biomedical Engineering Laboratory, Institute of Communication and Computer Systems, National Technical University of Athens, Athens, Greece; 2; 3 Dept. of Sports and Exercise Science (DISMOT), University of Palermo, Italy; 4 Pharmaceutical and Chemistry and Technologies, University of Padova, Italy; 5 Tisanoreica Research Center, Lonigo, Vicenza, Italy

## Background

Very low carbohydrate ketogenic type diets (VLCKD) have been shown to be more effective for body weight reduction and fat loss compared to balanced or low-calorie Mediterranean diets, at least in the short-medium term [1,2], although the underlying mechanisms of its efficacy are still not well understood. Despite being a diet in widespread use there are few data available regarding effects on respiratory ratio (RR) [3,4] and resting energy expenditure (REE) and, more specifically, there are no reports about the effects on RR following a return to a non ketogenic diet. The aim of this study was to compare the effects of a 20 day ketogenic Mediterranean diet with phytoextracts (KEMEPHY) and a low-calorie Mediterranean diet (MD) on RR and REE during and 20 days after finishing the ketogenic phase.

## Materials and methods

Forty healthy, overweight subjects were recruited and randomly divided into two groups: MD (age 46.61 ± 14.6, BMI 26.8±2.6, weight 76.3±9.9 kg ) and KEMEPHY (age 50.63 ±11.6, BMI 28.8±2.8, weight 81.8±11.6 kg). KEMEPHY group followed a ketogenic diet (<30g/day of carbohydrates) using meals that mimic the aspect and the taste of carbohydrates but with virtually zero CHO and with phytoextracts (Tisanoreica^®^, Lonigo, Italy); after 20 days of strictly KD subjects followed a low carbohydrate non ketogenic diet for 20 days. The MD group followed a standard low-calorie Mediterranean diet (tot Kcal). REE and RR, together with body weight and body composition, were measured in the morning after overnight fasting at the start of the study and after 20 (t_20_) and 40 days (t_40_). An Anova test for repeated measures and unpaired t-test with Welch’s correction were performed when appropriate.

## Results

Compared to starting values RR was significantly decreased in the KEMEPHY group after 20 days (p<0.05) and after 40 days (p=0.0002) (0.86±0.06; 0.79±0.05; 0.76±0.08; respectively) whilst no significant differences were detected in RR in the MED group. No significant differences in REE were detected. Both groups showed a significant decrease in body weight at t_20_ and t_40_ compared to basal conditions (KEMEPHY basal 81.8±11.6; t_20_ 77.8±11.4; t_40_ 75±11.2. MED basal 76.3±9.9; t20 75.6±9.9; 71.7±9.8) with the percentage changes in body weight being significantly greater for the KEMEPHY group. Both groups showed a significant decrease in body fat mass at t_20_ and t_40_ compared to basal conditions with the percentage changes in body fat mass being significantly greater for the KEMEPHY group (P=0.0135).

## Conclusions

These preliminary data showed that whilst both diet protocols lead to a significant decrease in body weight, the reduction was significantly greater during KEMEPHY. The KEMEPHY diet also lead to a lowering of RR and increased fat oxidation at rest without any effect on REE. These findings suggest that one of the main weight loss mechanisms of KD might be attributed to an improvement in resting nutrient oxidation and interestingly this effect was long lasting, at least for up to 20 days following cessation of the ketogenic. Data on metabolic effects of KEMEPHY 3 months the ketogenic period will soon be available.
